# Development of a versatile oncolytic virus platform for local intra-tumoural expression of therapeutic transgenes

**DOI:** 10.1371/journal.pone.0177810

**Published:** 2017-05-18

**Authors:** Nalini Marino, Sam Illingworth, Prithvi Kodialbail, Ashvin Patel, Hugo Calderon, Rochelle Lear, Kerry D. Fisher, Brian R. Champion, Alice C. N. Brown

**Affiliations:** PsiOxus Therapeutics Ltd, 154B Brook Drive, Milton Park, Abingdon, Oxfordshire, United Kingdom; Swedish Neuroscience Institute, UNITED STATES

## Abstract

Oncolytic viruses which infect and kill tumour cells can also be genetically modified to express therapeutic genes that augment their anti-cancer activities. Modifying oncolytic viruses to produce effective cancer therapies is challenging as encoding transgenes often attenuates virus activity or prevents systemic delivery in patients due to the risk of off-target expression of transgenes in healthy tissues. To overcome these issues we aimed to generate a readily modifiable virus platform using the oncolytic adenovirus, enadenotucirev. Enadenotucirev replicates in human tumour cells but not cells from healthy tissues and can be delivered intravenously because it is stable in human blood. Here, the enadenotucirev genome was used to generate plasmids into which synthesised transgene cassettes could be directly cloned in a single step reaction. The platform enabled generation of panels of reporter viruses to identify cloning sites and transgene cassette designs where transgene expression could be linked to the virus life cycle. It was demonstrated using these viruses that encoded transgene proteins could be successfully expressed in tumour cells *in vitro* and tumours *in vivo*. The expression of transgenes did not impact either the oncolytic activity or selective properties of the virus. The effectiveness of this approach as a drug delivery platform for complex therapeutics was demonstrated by inserting multiple genes in the virus genome to encode full length anti-VEGF antibodies. Functional antibody could be synthesised and secreted from infected tumour cells without impacting the activity of the virus particle in terms of oncolytic potency, manufacturing yields or selectivity for tumour cells. *In vivo*, viral particles could be efficaciously delivered intravenously to disseminated orthotopic tumours.

## Introduction

The first oncolytic virus therapy has now been approved in the USA and Europe for the treatment of cancer. Talimogene laherparepvec (T-Vec, Imlygic®), an oncolytic herpes simplex virus, was approved in late 2015 for the treatment of metastatic melanoma having shown improved durable response rates in patients with unresectable stage IIB to IV disease [1, 2]. Following in the wake of this success a broad spectrum of oncolytic viruses, using RNA, DNA, non-enveloped or enveloped viral vectors are currently being clinically evaluated for cancer treatment (Reviewed in [3–5]).

Oncolytic viruses were originally developed for their ability to infect, replicate and directly kill human tumour cells while being attenuated in normal cells [6]. Their proposed primary mechanism of action was that locally delivered virus particles would rapidly spread and destroy tumours while having limited off-target activity in surrounding healthy tissue. More recently however, data emerging from both pre-clinical and clinical studies has led to a shift in the understanding of how oncolytic viruses illicit their anti-tumour activity by demonstrating a key role of the immune system in the mechanism of action of effective virus therapy (Reviewed in [5, 7]). In particular, studies have shown oncolytic virus activity mediates increased T cell infiltration and anti-tumour immune responses in animal models [8–11]. In the clinic, immune mediated regression has been observed in uninjected distal tumours, which is thought to be mediated by an induced anti-tumour immune response [12, 13]. These data have led to oncolytic viruses emerging as a novel class of agent in an expanding group of cancer therapies known collectively as immuno-therapies.

Cancer Immunotherapies target pathways which regulate immune responses to tumour antigens by either directly activating the innate or adaptive immune system or by blocking pathways that mediate immune suppression [14]. In the clinic, cancer immunotherapies have recently demonstrated profound and durable immune-mediated efficacy in patients with previously untreatable metastatic disease [15, 16]. This has been most dramatically demonstrated using antibodies targeted against molecular checkpoints of T cell activation [17–19].

Although the clinical success of immunotherapies has led to a step-change in the treatment of cancer, when used as monotherapies, responses are still limited to a subset of patients and a subset of cancer types [20, 21]. Currently, combinations of two or more agents that target different mechanisms have demonstrated a clear improvement in response rates, but have also highlighted several key challenges in systemically delivering multiple immunotherapeutics [22–24]. Firstly, enhanced efficacy is often accompanied by an increased rate and severity of drug-related adverse events caused by off-target toxicity [25, 26]. Secondly, providing treatment with two or more complex biological agents is associated with high costs [27]. To overcome such challenges, novel single-agents that can safely deliver multiple therapeutics to tumours are required.

Oncolytic viruses could potentially provide a unique solution to this through encoding within the virus genome one or more therapeutic transgene which can synergise with the viruses’ oncolytic and immunostimulatory mechanisms of action. The concept of using viral vectors as drug delivery platforms is not new, however the majority of oncolytic and other viral vectors, including T-Vec, are not useful as systemic delivery platforms because they are neutralised by blood components and therefore can only be delivered intratumourally to patients [28, 29]. To address this limitation Kuhn *et al*. took a novel approach to generating oncolytic viruses by serial passaging pools of adenovirus serotypes on colon carcinoma cells and then selecting for viruses with potent and selective oncolytic activity [30]. The lead oncolytic adenovirus generated by this process was enadenotucirev (formerly ColoAd1), which is a serotype B Ad11p/Ad3 chimeric adenovirus. This virus showed potent oncolytic activity, an ability to be released and spread from tumour cells prior to lysis and tropism for a broad range of epithelial-derived tumours. Pre-clinical studies have also demonstrated enadenotucirev is stable and infectious in the presence of human whole blood, blood cells and serum immunoglobulins [31]. Enadenotucirev, has now undergone clinical evaluation and delivery of enadenotucirev virus particles to tumours following intravenous dosing has been demonstrated in patients through detection of both viral DNA and virus proteins in tumour tissue [32]. These data clearly indicated that enadenotucirev had the potential to be a systemic drug delivery platform if therapeutic genes could be readily encoded in the virus genome without disturbing viral properties.

Non-replicating adenovirus vectors, based on adenovirus type 5, are routinely used in laboratory applications for the introduction of transgenes into cells, and have been successfully ‘armed’ to encode transgenes for therapeutic gene delivery [33]. Adenoviruses make good platforms for encoding exogenous genes because they have double stranded DNA genomes that are inherently stable and which don’t suffer from the rapid mutation rates faced by RNA viruses. However, adenovirus particle stability can be compromised if genome size is increased by > 5%, thereby limiting their packaging capacity [34]. Enadenotucirev is unlikely to have these packaging limitations due to deletions that occurred in the virus genome during the bioselection process [30]. The enadenotucirev virus has a capsid identical to its parental virus Ad11p but a genome 2470bp shorter than Ad11p due to deletions in the regions coding for the early genes, E3 and E4. These genome characteristics combined with its ability to be systemically delivered therefore made enadenotucirev an attractive candidate for ‘arming’ with therapeutic genes. However, to develop a virus into an effective drug delivery platform methods for efficiently and stably inserting transgenes into the genome are required.

The most commonly used approach for inserting transgene cassettes into adenovirus vectors is by homologous recombination in *E*.*coli*. This process requires multiple cloning and amplification steps which are time consuming, often inefficient and introduce a risk of having multiple DNA species in the final material. Alternatively transposon based systems have been used for random insertion of transgene cassettes into adenovirus genomes. While using transposons is useful for identifying potential genomic sites for transgene insertion, it is not an efficient method to generate viruses to optimise transgene cassette design. Both recombination and transposon approaches are therefore not appropriate when engineering adenovirus genomes for the generation of drug development candidates, where rapid production of well characterised virus panels for each therapeutic target of interest is required. The purpose of this work was therefore to develop a novel and versatile molecular biology system, based on enadenotucirev, for the rapid generation of panels of candidate viruses. These viruses were used to optimise the platform such that one or more functional transgene could be expressed without altering the anti-tumour activity of the enadenotucirev virus.

## Results

### Platform overview and enadenotucirev vector construction

**Fig 1:**
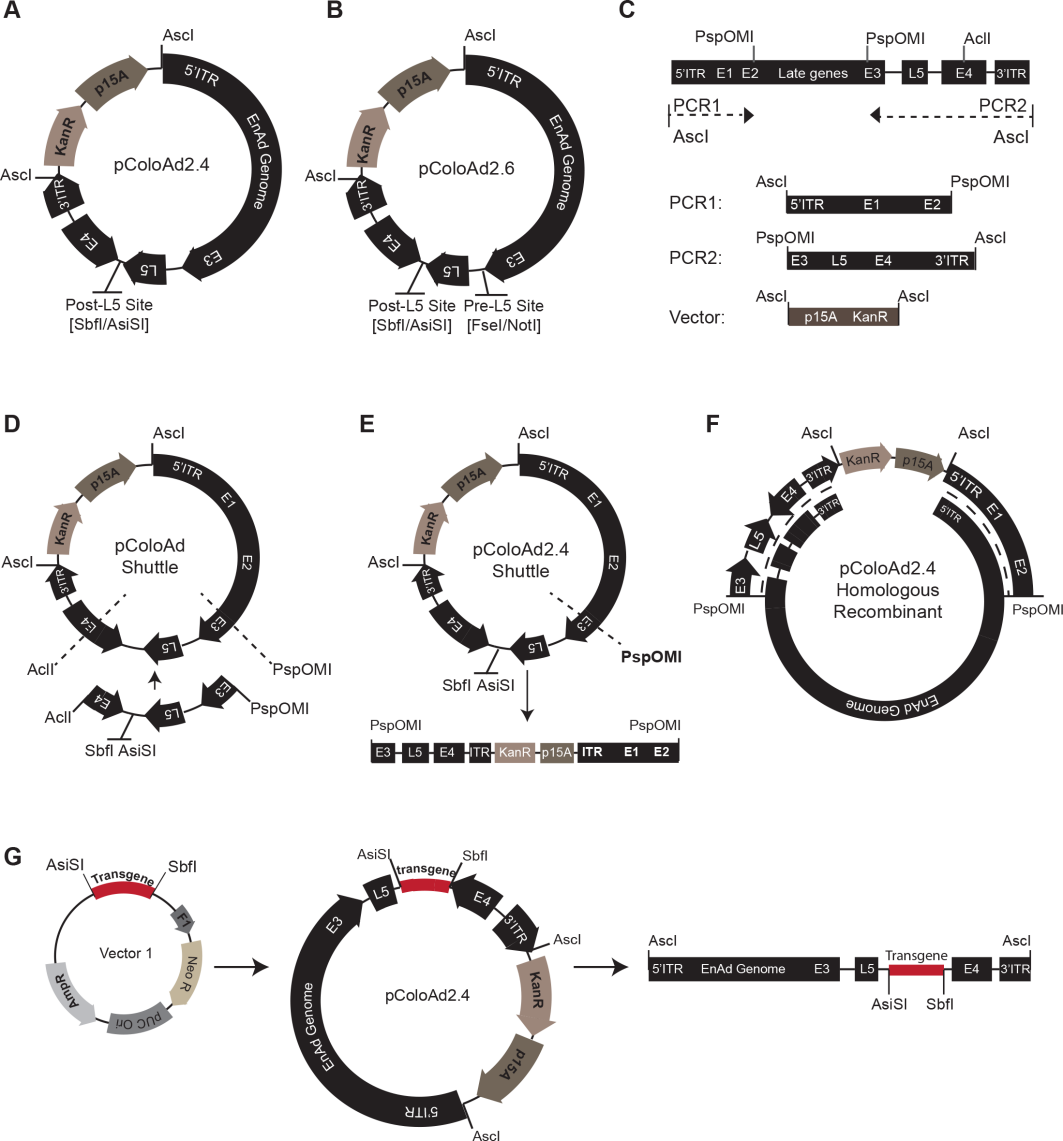
Enadenotucirev vector construction and transgene cloning. (A) Plasmid map for the pColoAd2.4 vector and (B) plasmid map for the pColoAd2.6 vector. (C) Schematic showing the two PCR products generated from the enadenotucirev linear genome and a synthetically generated p15A linearised vector. (D) Plasmid map of the pColoAd shuttle generated by three way ligation of the two PCR products and p15A vector shown in C. AclI and PspOMI sites indicated (dotted lines) were restriction digested to allow replacement of the interlinking DNA with a synthetically generated equivalent sequence containing AsiSI and SbfI sites. The vector generated by this replacement is pColoAd2.4 shuttle. (E) Plasmid map of the pColoAd2.4 shuttle vector and map of the vector post-linearisation with the enzyme PspOMI. (F) Schematic showing the sites of homologous recombination (dotted line) between the linearised pColoAd2.4 shuttle vector and the enadenotucirev linear genome to generate the pColoAd2.4 vector. (G) Schematics showing how transgene cassettes are directly ligated from a sub cloning vector (‘vector 1’) into the pColoAd2.4 vector between AsiSI and SbfI sites. The plasmid generated is then digested with AscI to rescue the enadenotucirev linear virus genome encoding the transgene(s).

We set out to generate a cloning platform that would permit single-step direct ligation of therapeutic transgene cassettes into the enadenotucirev genome, avoiding the need for recombination for transgene insertion. To this end two novel plasmids were generated that contained the enadenotucirev genome with either two introduced unique restriction enzyme sites recognised by the enzymes AsiSI and SbfI (pColoAd2.4, Fig 1A) or 4 unique restriction sites recognised by the enzymes NotI, FseI, AsiSI and SbfI (pColoAd2.6, Fig 1B). In both plasmids the unique restriction sites for AsiSI/SbfI were located towards the 3’ end of the enadenotucirev genome between the polyA recognition site for the L5 gene (Fibre) and the polyA recognition site of the E4 gene region; this site is designated ‘post-L5’ site. The unique restriction sites for NotI/FseI in pColoAd2.6 were located in the region between the stop codon of the E3 gene and start codon of the L5 gene, this site is designated the ‘pre-L5’ site. These 3’ regions were specifically selected for the insertion of transgene cassettes because they are in non-coding regions of the enadenotucirev genome and located downstream of endogenous promoters responsible for modulating genes controlling the virus life cycle and capsid assembly.

To generate the pColoAd2.4 plasmid the enadenotucirev viral genome was homologously recombined in *E*.*coli* with a linearised novel shuttle vector, pColoAd2.4 Shuttle. The construction of pColoAd2.4 shuttle and pColoAd2.4 is summarised in Fig 1C–1F and described in detail in the Materials & Methods. The pColoAd2.6 plasmid was generated from chemically synthesised DNA oligos by Gibson assembly, thereby overcoming any need for recombination in *E*.*coli* during the vector construction process. Both plasmids were stable when transformed into *E*.*coli*, and could be readily amplified and purified by standard plasmid purification methods.

The pColoAd2.4 vector was used to establish an efficient method for the directional insertion of transgene cassettes into the vectors. The ligation strategy developed for the cloning of transgene cassettes and the method for excising purified genomes for viral production is outlined in Fig 1G and is described in detail in the Materials and Methods. Briefly, the transgene cassette of interest was synthesised or sub-cloned into a small plasmid that contained an ampicillin resistance cassette. The transgene cassette was then directly inserted into the enadenotucirev genome in 1-step by AsiSI and SbfI restriction of the pColoAd2.4 and the sub cloning vectors, followed by overnight ligation. Ligation products were amplified in *E*.*coli* plated on kanamycin plates and successful production of pColoAd2.4 vectors containing transgenes was confirmed by restriction analysis and sequencing. The modified viral genome could then be excised from the vector by AscI digestion and used for virus production in an appropriate cell line. Using this method a ligation efficiency of between 20%-100% (n = 35 transgenes tested) could be obtained. The precise ligation conditions to obtain this efficiency were determined following detailed investigation of the relative amounts of transgene to insert in the ligation reaction, the ligation time, temperature and the *E*.*coli* strain. Interestingly, ultra-competent cell strains such as XL Golds were less efficient for both transformation and amplification of constructs than standard highly competent cloning strains such as XL-1. XL-1 produced high plasmid yields following initial transformation such that further rounds of amplification were not required in order to produce sufficient plasmid yields for virus production, this significantly reduced the time required to generate viral genomes. Following extensive use of this platform, it has been found that cloning efficiency is also related to the length of the inserted transgene with transgene cassettes greater than 3kb having a decreased cloning efficiency. The optimised conditions have now been successfully used to clone a range of transgene cassettes of 0.7kb-2kb (mean efficiency 55 ± 18%), 2kb- 3kb (mean efficiency 55 ± 26%) and >3kb in length (mean efficiency 28 ± 7%). Importantly these conditions produced a reproducible and efficient method for cassette insertion that does not require the use of selectable markers in the transgene cassettes (such as antibiotic resistance genes). This approach therefore avoids the introduction of unwanted genes into downstream therapeutic viruses, maximises the available space for encoding therapeutics and permits rapid production of viral genome panels for testing.

### Platform exemplification using reporter genes

**Fig 2:**
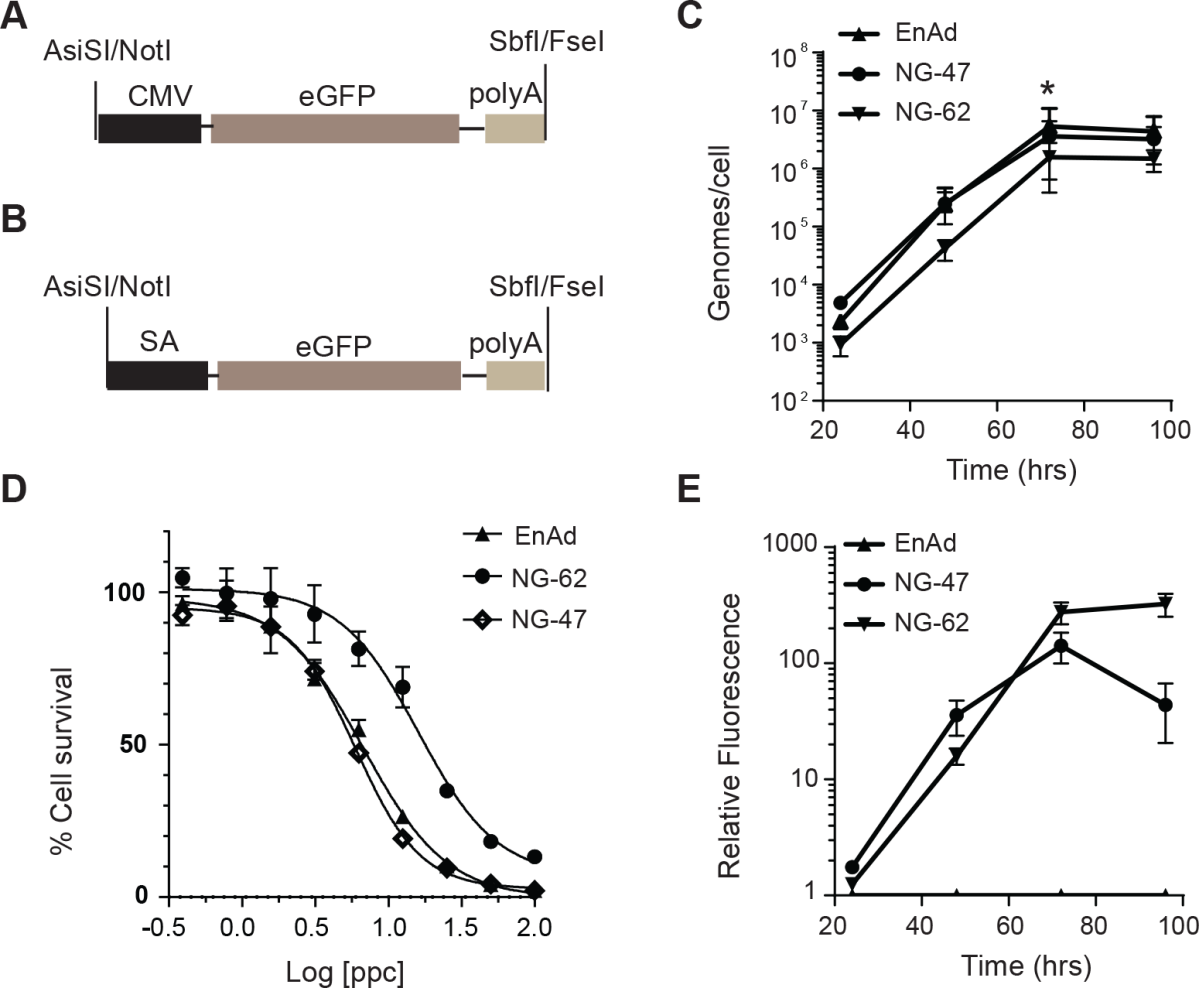
Reporter transgene expression mediated by endogenous and exogenous promoters. (A) Schematic of the GFP expression cassette encoded in the NG-47 and NG-274 viruses. (B) Schematic of the GFP expression cassette encoded in the NG-62 and NG-252 virus. (C) The total genomes generated per HT-29 colon carcinoma cell infected for 24–96 hrs with 1 particle per cell (ppc) NG-47, NG-62 or enadenotucirev (EnAd) virus particles. Graph shows mean ± SD (n = 3 independent experiments, *P<0.05). (D) HT-29 cells infected with enadenotucirev, NG-47 or NG-62 virus particles over a range of 100–0.39 ppc for 72hrs then assessed for cell viability by MTS assay. Graph shows quantification of % cell survival, defined by mitochondrial activity, relative to uninfected controls. (E) Quantification of total accumulated GFP fluorescence in wells containing HT-29 cells infected, as in (C), for 24-96hrs with 1ppc of enadenotucriev, NG-47 or NG-62 virus particles. Graph shows the fold change in fluorescence at each time point relative to uninfected control cells plotted as mean ± SD (n = 3 independent experiments), no signal could be detected at any time point in enadenotucirev infected cells.

Having established an efficient cloning method we determined whether enadenotucirev genomes containing transgenes could be used to generate functional viruses. It had been demonstrated previously using transposon based systems in Ad5 that insertion of cassettes in some 3’ regions of the Ad5 genome was compatible with transgene expression using either exogenous promoters, such as CMV, or promoters endogenous to the virus [35]. In particular these data demonstrated that by utilising human-specific splice acceptor sequences transgenes could be expressed at high levels when under the control of the Ad5 major late promoter (MLP). To investigate whether the MLP could be used to control transgene expression in enadenotucirev we designed two cassettes encoding the green fluorescent protein, eGFP. The first contained eGFP cDNA preceded by a CMV promoter and the second eGFP cDNA preceded by a splice acceptor (SA) sequence (Fig 2A and 2B respectively). The cassettes were introduced into the enadenotucirev genome at either the post-L5 site of pColoAd2.4 (viruses designated NG-47 [CMV] and NG-62 [SA]) or the pre-L5 site of pColoAd2.6 (viruses designated NG-274 [CMV] and NG-252 [SA]). Cloning efficiency of transgene cassettes was similar for all viruses. Production of the virus material from the DNA genomes was then attempted in HEK293 cells. Although enadenotucirev viruses are fully replication competent, HEK293 cells were used to produce virus particles due to the availability of established cGMP production protocols. Transfection of the four modified genomes into HEK293 cells yielded active viruses as verified by observation of significant cytopathic effects (CPE) in the cell monolayers. However, functional transgene expression could only be observed by fluorescence microscopy when eGFP was under the control of a CMV promoter, or when a splice acceptor containing cassette was located in the post-L5 site. Cassettes located in the pre-L5 site did not yield any observable GFP fluorescence when a splice acceptor sequence was used. This was initially surprising because in Ad5 a corresponding site has been shown to be compatible with high levels of transgene expression using endogenous viral promoters [35]. However, in contrast to Ad5, little is known about the regulatory elements controlling gene expression in enadenotucirev (or class B adenoviruses in general) or how gene regulation in this region of the enadenotucirev genome may have been altered by the 2445bp deletion in the E3 gene. Additional analysis of mRNA species would be required to fully understand why this site is not compatible with detectable gene expression using endogenous promoters.

Further platform optimisation was therefore focused on the post-L5 site and characterisation of virus and transgene activity was carried out using the viruses, NG-47 and NG-62. During production of the NG-47 and NG-62 virus material in HEK293 production cell lines it was observed that the total particle yield for NG-62 was slightly lower than for NG-47 (6.8e11vp vs 1e12vp, respectively). After initial production and observation of the viruses in the HEK293 manufacturing cells, we went on to characterise virus replication, potency and GFP production in HT-29 colon carcinoma cell lines. The kinetic of virus genome production, up to 96hrs post-infection was equivalent for all three viruses, with genome production, quantified in both the cellular supernatant and cell lysate, peaking at 72hrs (Fig 2C). At this point infected, dying cells, could be observed in all virus treated wells and hence no further increase in genome production was detected between 72 and 96 hrs. However, analysis of the total genome yield at each time point revealed that NG-62 produced somewhat fewer genomes at each timepoint and had significantly lower genome production at 72hrs (4.73e6 genomes per cell) compared to either NG-47 or enadenotucirev (1.1e7 and 1.6e7 genomes per cell, respectively). Similarly, assessment of oncolytic potency of the viruses using a MTS assay, which assesses the metabolic activity of cellular mitochondria as an indicator of cell viability, demonstrated a reduction in oncolytic potency of NG-62 (EC50 of 16ppc) compared to NG-47 or enadenotucirev (EC50 of 5ppc and 7ppc respectively) (Fig 2D).

In parallel to assessing virus genome replication (Fig 2C) we also compared the accumulation of GFP in HT-29 culture wells using a plate assay to quantify total fluorescence (Fig 2E). These data showed that GFP accumulated at slightly higher levels in wells containing NG-62 infected cells than NG-47 infected cells. These data also indicated that highest levels of NG-62 mediated GFP fluorescence accumulated late in the virus life cycle consistent with expression being mediated by the major late promoter.

Collectively these data demonstrated that transgene expression could be successfully driven by the enadenotucirev endogenous MLP and total transgene yield from the MLP is at least as high as that driven by a CMV promoter. However, this transgene expression driven by the MLP appeared to be associated with a reduction in virus activity in terms of oncolytic activity and total genome replication.

### Platform optimisation by modification of transgene cassette design

In adenoviruses the MLP drives transcription of the major late transcription unit which encodes the capsid proteins essential to the formation of infectious virus particles. The differential expression of these capsid proteins is regulated both transcriptionally and post-transcriptionally by alternative splicing and alternative polyadenylation [36]. One explanation for the decreased viral activity and high transgene yield observed for the NG-62 virus was that the exogenous splice acceptor and polyadenylation sequences encoded in the GFP cassette mediated preferential translation of the GFP protein over endogenous virus proteins required for functional virus production.

**Fig 3:**
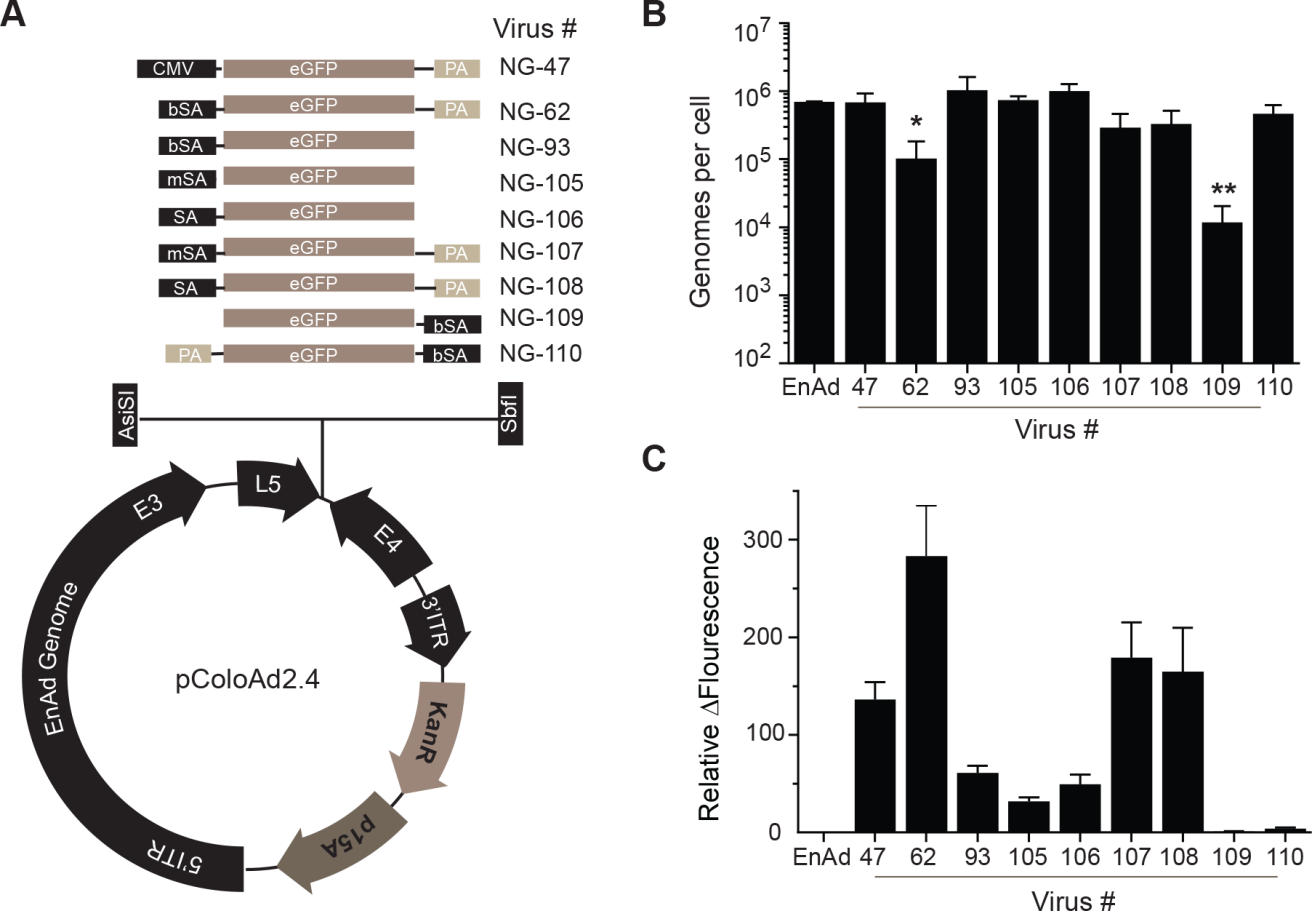
Characterisation of gene expression and viral activity *in vitro* using a GFP reporter virus library. (A) Schematics of GFP reporter virus transgene cassettes inserted into the pColoAd2.4 vector. (B) Viral genomes detected in the supernatants of HT-29 cells infected for 72hrs with 1ppc of enadenotucirev or GFP encoding viruses quantified by qPCR. Graph shows genomes produced per cell plotted as mean ± SD (n = 3 independent experiments, *P<0.05, **P<0.005 one-way Anova). (C) Quantification of GFP fluorescence in HT-29 cells infected as in (B). Graph shows change in fluorescence relative to uninfected control cells plotted as mean ± SD (n = 3 independent experiments).

We therefore set out to determine if altering these encoded regulatory elements could result in a more effective balance between transgene production and virus activity. Initially we used transgene cassettes encoding green fluorescent protein (GFP) as this reporter transgene could be used to rapidly provide information on transgene and virus activity (Fig 3). A panel of GFP encoding transgene cassettes was generated that contained a 5’ branched splice acceptor sequence–TGCTAATCCTTTCTCTCTTCAGG- (bSA; NG-62, NG-93), a splice acceptor sequence -TTTCTCTCTTCAGG- (SA; NG-106 and NG-107) or a minimal splice acceptor sequence–CAGG- (mSA; NG-105 and NG-108) (Fig 3A). For each splice acceptor cassette a poly(A) sequence was either included at the 3’ end of the GFP cDNA or not. Cassettes were also designed in a reverse orientation to investigate if transgene expression could be mediated by the endogenous E4 promoter located at the 3’ end of the enadenotucirev genome (NG-109, NG-110). Virus activity and transgene expression was assessed in parallel in the colon carcinoma cell line, HT-29, 72 hours post-infection. Virus replication was measured by quantifying the amount of viral genome in the culture supernatants (Fig 3B) and GFP production was assessed by quantifying total fluorescence in the culture wells (Fig 3C). Analysis of replication was performed on supernatant samples because enadenotucirev, like some other non-enveloped viruses, is released from infected cells both pre- and post-lysis, although the mechanisms mediating pre-lysis release have yet to be elucidated [30, 37]. The NG-62 virus, as previously characterised, showed reduced replication compared to enadenotucirev or NG-47 (NG-62, 9x10^4^ genomes/cell compared to 6.3x10^5^ or 6.2x10^5^ genomes/cell, respectively) under these conditions. Importantly, this defect in replicative ability could be recovered by removal of the poly(A) tail from the transgene cassette (NG-93, 9x10^5^ genomes/cell) or by replacement of the branched splice acceptor with either a splice acceptor (SA) (NG-106/108) or minimal splice acceptor (mSA) (NG-105/107). When the SA or mSA was used replication was similar (8.9x10^5^ vs 3.0x10^5^ genomes/cell) or (6.7x10^5^ vs 2.6x10^5^ genomes/cell) whether a poly(A) tail was included in the cassette or not. In contrast when cassettes using a bSA were in the reverse orientation virus replication was equivalent to enadenotucirev (4.1x10^5^ genomes/cell) when a poly(A) sequence was encoded but was severely compromised (1x10^4^ genomes/cell) when the poly(A) sequence was not encoded.

Assessment of transgene expression showed that inclusion of a poly(A) sequence in the transgene cassette significantly enhanced GFP expression independent of which splice acceptor sequence was used. The lower level of GFP detected following infection with viruses lacking the poly(A) tail was not due to a difference in virus lytic activity as virus replication and the peak of GFP fluorescence accumulation was equivalent (S1 Fig). In addition, these data revealed that using the MLP (e.g. NG-62) resulted in significantly higher levels of GFP expression (283 fold change over control cells) than using the early E4 promoter (3.8 fold change over control cells) even though the NG-110 and NG-62 viruses had equivalent viral genome replication.

Taken together these data demonstrated that by modulating transgene cassette design the adenovirus major late promoter can be effectively used to produce transgene yields equivalent to or higher than those from an exogenous promoter such as CMV, while still maintaining the replication characteristics of the parental virus. Furthermore, by using poly adenylation or alternate splicing sequences the level of transgene expression can be modulated without attenuating the virus or altering virus genomic characteristics.

### Selective transgene expression and virus delivery *in vivo*

To demonstrate the flexibility of the developed platform and to permit in depth characterisation of its potential for selective delivery of functional transgenes to tumours, we cloned viral genomes encoding transgene cassettes for the reporter enzyme *firefly* luciferase. Three genomes containing luciferase cassettes were generated, one using a CMV promoter (NG-61) and the others using the splice acceptor sequences, bSA (NG-63) or mSA (NG-285). Following transfection of the genomes into HEK293 cells significant CPE could be observed within 168 hrs for the NG-61 and NG-285 viruses. For the NG-63 virus, although some lytic cell death could be observed, a significant productive infection could not be detected. This observation correlated with the results seen for the bSA-GFP encoding virus NG-62, which showed significantly reduced virus activity compared to CMV or mSA containing viruses. One explanation for the more profound dysfunction in virus activity for NG-63 than NG-62 could be that luciferase is a larger, more complex protein than GFP and high expression under bSA regulation resulted in increased cellular stress preventing virus particle production.

**Fig 4:**
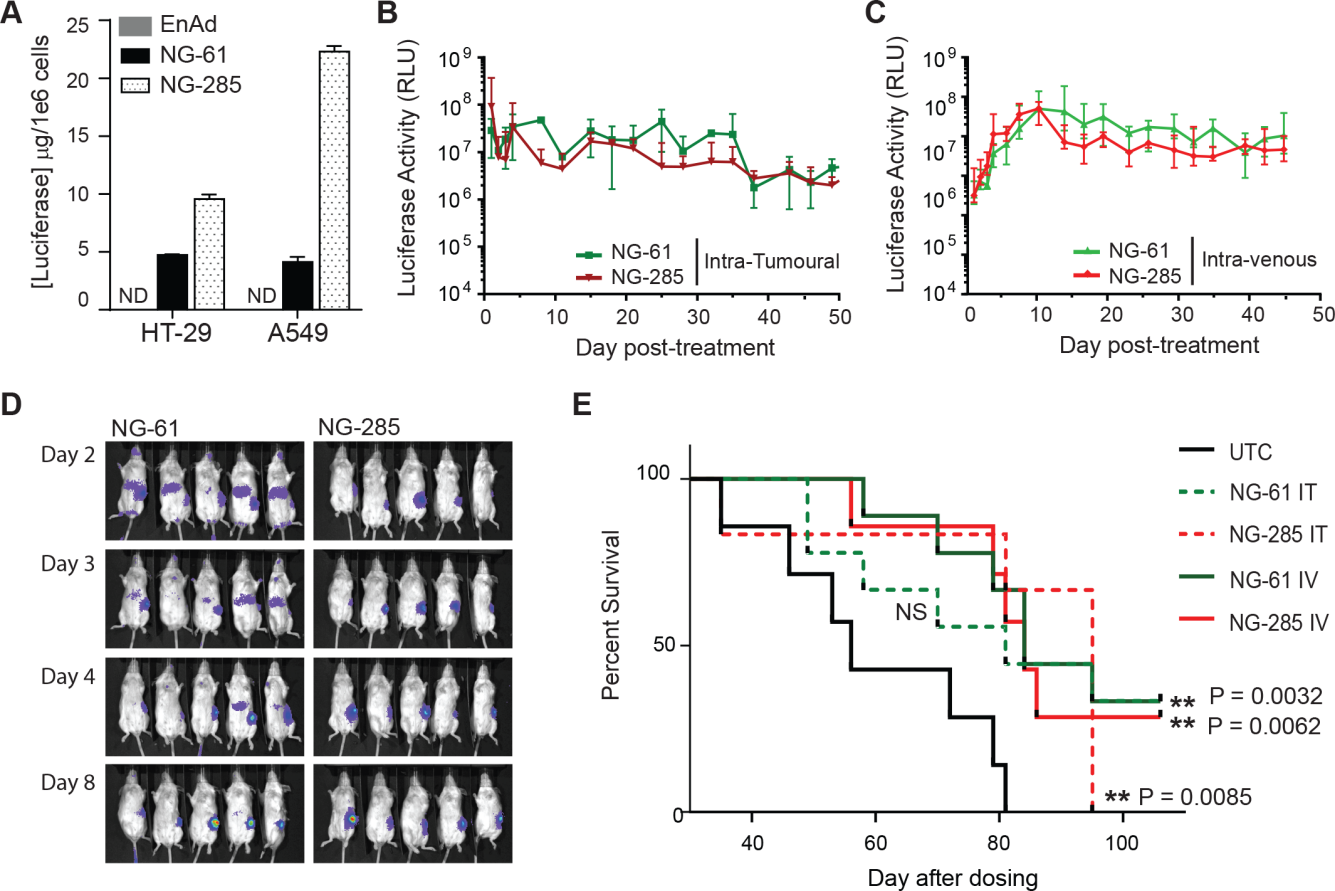
Characterisation of transgene expression, virus activity and virus efficacy *in vivo* using luciferase reporter viruses. (A) Quantification of luciferase expression (μg/1x10^6^ cells) in HT-29 and A549 cells infected with 10 ppc enadenotucirev, NG-61 or NG-285 virus particles for 24-48hrs. (B) Measurement of luciferase activity by imaging A549 lung carcinoma sub-cutaneous xenograft tumours treated IT at Day 0 with either 5x10^9^ NG-61 or NG-285 virus particles. (C) Measurement of luciferase activity by imaging A549 lung carcinoma sub-cutaneous xenograft tumours treated IV at Day 0 with either 5x10^9^ NG-61 or NG-285 virus particles. Graphs shows light units detected at each time point relative to untreated controls, data is plotted as relative light units (RLU) median ± interquartile range for n = 5 mice per treatment group. (D) Images showing detection of luciferase expression in mice bearing A549 subcutaneous xenograft tumours (as in (C)) treated IV with 5x10^9^ NG-61 (left panels) or NG-285 (right panels) virus particles. Luciferase expression could be observed in flank tumours in all treated mice and in the livers of NG-61 treated mice (left panel, Day 2–4). (E) Kaplan-Meier plot showing percent survival, assessed by measurement of tumour burden, for mice imaged in B-D that were bearing A549 subcutaneous xenograft tumours treated with 5x10^9^ NG-61 or NG-285 virus particles delivered by single IT or single IV injection at Day 0 (n = 10 mice per treatment group, **P<0.005 Log rank test).

Further characterisation was therefore carried out using NG-61 and NG-285 viruses. Amplification and purification of NG-61 and NG-285 revealed similar particle yields. HT-29 cells, used to characterise the GFP expressing viruses (Figs 2 and 3), are one of a broad panel of carcinoma cell lines in which enadenotucirev has been previously shown to be active [30]. We therefore demonstrated NG-61 and NG-285 virus activity in both HT-29 cells and a second carcinoma cell line, A549, which could also be used to investigate virus activity in murine models. Luciferase transgene protein could be detected in both cell lines and was higher when controlled by the endogenous major late promoter compared to a CMV promoter (Fig 4A).

To establish the functional activity of the viruses *in vivo*, virus and transgene activity was investigated in immunodeficient (*CD1-nude*) mice bearing sub-cutaneous human lung carcinoma tumours (A549). Mice were dosed either IT or IV with NG-285 or NG-61 viruses and monitored for transgene activity in the tumour and other organs by luminescence imaging and virus efficacy by assessing survival via measuring tumour volume.

Transgene expression could be detected in the tumours of all NG-61 and NG-285 IV-treated animals after 24 hrs, although at lower levels than those observed by IT delivery, indicating only a portion of the virus dose reached the tumour by the IV delivery route (NG-285, 9.2x10^8^ RLU vs 3.3x10^6^ RLU) and (NG-61, 2.9x10^8^ vs 3.1x10^6^ RLU). However, consistent with the mechanism of action of an oncolytic virus, transgene expression levels in the IV cohort then steadily increased up to day 10 post-treatment before plateauing and maintaining similar levels of expression to those measured for IT delivery (Fig 4E and 4F and S2 Fig). These data showed that oncolytic viruses can close the initial delivery gap between IT and IV dosing via *in situ* replication.

Bioluminescence imaging was only carried out up to day 49 of the study because at this point the untreated control mice reached their survival endpoint in terms of tumour volume. Up to this point, luciferase expression was comparable between the two viruses, however off-target expression in the liver was significantly different. NG-61 treated mice had detectable transgene expression in the liver within 24hrs of IV dosing (Fig 4G) which declined over the subsequent 7 days becoming no longer detectable by Day 8. This data indicated that a transient transduction of cells in the liver may permit CMV driven gene expression as this does not require active virus replication or amplification. This short lived transgene expression in the liver also correlated with previous pre-clinical work using enadenotucirev that showed primary liver cells are not permissive to viral replication, late gene expression or particle production [30]. Importantly, and consistent with a lack of active infection in the liver, no transgene expression could be detected at any time point post IV dosing with the NG-285 virus (Fig 4G). These data therefore demonstrated that by linking transgene expression to the activity of the virus’ MLP the tumour selectivity of enadenotucirev can be conferred to transgene expression.

Viral efficacy, monitored by survival to a pre-determined maximum tumour volume of 1200mm^3^ in the subcutaneous A549 lung carcinoma model, demonstrated a significant increase in survival post-treatment for both NG-61 and NG-285 animals treated IV and for NG-285 treatment IT (Fig 4H). This further demonstrated that prolonged transgene production using the enadenotucirev platform can be mediated *in vivo* in the context of an efficacious oncolytic infection following IV delivery.

### Expression of complex therapeutic proteins

Having established enadenotucirev as a readily modifiable platform for selective transgene expression in tumours, a next objective was to determine if the platform would be broadly effective for expressing complex or multiple therapeutic genes. To this end we selected full length monoclonal antibodies as a secreted protein product by which to test the platform. A genome (NG-135) was generated that encoded the sequence for the heavy and light chains of a humanized monoclonal IgG1 antibody to human VEGF flanked by a mSA sequence and poly(A) tail. In this genome the heavy and light chain coding sequences were linked by an IRES sequence and leader sequences were included at the start of the heavy and light chain transgenes to mediate secretion of antibody from virally infected tumour cells prior to and without the need for oncolysis. A schematic of the transgene cassette is shown in S3 Fig.

**Fig 5:**
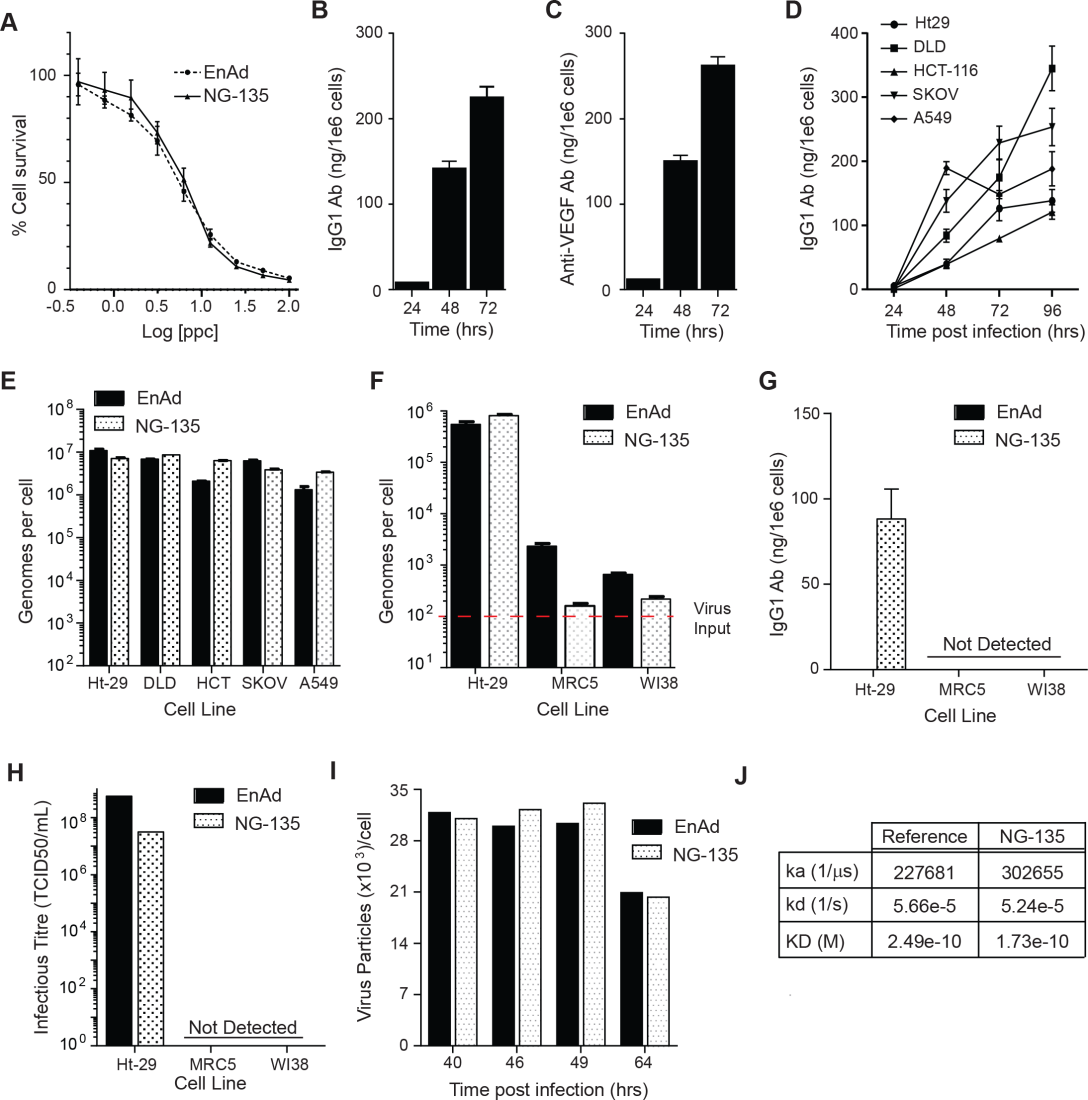
Characterisation of virus activity and functional expression of monoclonal antibodies. (A) HT-29 cells infected with NG-135 or enadenotucirev virus particles over a range of 100–0.39 ppc for 72hrs then assessed for cell viability. Graph shows quantification of % cell survival relative to uninfected controls. (B) IgG1 antibody expression detected in cellular supernatants of HT-29 cells infected with 10ppc NG-135 by anti-IgG1 ELISA. (C) Quantification of VEGF-165 binding of antibody expressed in cellular supernatants described in (B). (D) IgG1 antibody expression, assessed by anti-IgG1 ELISA, in cellular supernatants of carcinoma cell lines infected for 24-96hrs with NG-135 at 1ppc. (E) Viral genomes (quantified by qPCR) in the supernatants of carcinoma cells infected for 96hrs with 1ppc of enadenotucirev or NG-135. (F) Viral genomes (quantified by qPCR) in the supernatants of HT-29 or fibroblast cell lines infected for 72hrs with 100ppc of NG-135 or enadenotucirev. (G) IgG1 antibody expression detected in cellular supernatants described in (F). (H) Infectious virus particle titre assess by TCID_50_ using cellular supernatants described in (F). (I) Total virus particle yield per cell (quantified by HPLC) in the cellular and supernatant culture fractions of HEK293 cells infected for up to 64hrs with NG-135 or enadenotucirev at 50ppc. (J) Table showing results obtained from Biacore analysis of VEGF-165 binding for antibody purified from NG-135 infected HEK293 cells compared to the bevacizumab reference standard. The data shows the equilibrium dissociation constant between the antibody and the VEGF antigen (KD), calculated from the rates of antibody to antigen association (Ka) and dissociation (kd). Unless otherwise stated all graph data points are plotted as mean ± SD (n = 3 experimental replicates).

Oncolytic activity and antibody expression was first characterised in NG-135 or enadenotucirev infected HT-29 carcinoma cells *in vitro*. Analysis of oncolytic potency by cytotoxicity assay demonstrated no loss in oncolytic activity for NG-135 compared to enadenotucirev (EC50’s 5.6 and 6.0, respectively, Fig 5A) and analysis of antibody expression by ELISA showed human IgG1 could be detected in the supernatants of NG-135 infected HT-29 cells at 24, 48 and 72hrs post-infection (Fig 5B). The secreted antibody was also detectable, at similar levels to the IgG1 ELISA, when using a VEGF binding ELISA in which supernatants were incubated with immobilised human VEGF and anti-VEGF antibody detected via the Fc portion (Fig 5C). Importantly, these data demonstrated that a full length therapeutic antibody could be successfully encoded as two separate gene products in the enadenotucirev genome.

Enadenotucirev is active against a wide range of epithelial-derived tumours, and has been investigated clinically in multiple indications including colorectal, ovarian, lung, bladder and renal carcinoma [32, 38, 39]. While enadenotucirev is broadly active in carcinomas it is also highly selective for human tumour cells of epithelial origin over healthy human non-tumour cells (primary or cell lines) or mouse tumour cells. We therefore determined whether NG-135 could produce antibodies in a range of cancer cell types while still maintaining the virus activity and selectivity properties of enadenotucirev. Genome replication and antibody expression was first analysed in a panel of carcinoma cell lines known to be permissive to enadenotucirev infection (colon carcinomas, HT-29, HCT-116, DLD-1, lung carcinoma, A549 and ovarian carcinoma, SKOV3). Antibody was detected in the supernatant of infected cells, and accumulated in the supernatant over the time course of viral infection (24 – 96hrs) for all cell lines tested (Fig 5D). The expression of antibody over the course of infection did not impact NG-135 viral replication as the total genomes detected by 96 hrs was similar to enadenotucirev for all cell lines (Fig 5E). To explore the selectivity of NG-135, virus activity and antibody production was next compared in two fibroblast cell lines (Wi38 and MRC5) known to be minimally-permissive to enadenotucirev infection. MRC5 or Wi38 cells incubated with NG-135 or enadenotucirev virus particles showed minimal detectable amplification of virus genomes in contrast to the HT-29 cells used as a permissive control cell line in the assay (Fig 5F). This correlated with a lack of detectable antibody expression in the cellular supernatant of either fibroblast cell line at 72hrs post treatment (Fig 5G). Based on the sensitivity limits of the ELISA this lack of detectable antibody represents at least a 2000-fold window of selectivity for transgene expression by tumour cells compared to non-tumour cells. Transfer of the culture supernatants on to permissive HT-29 cells for a further 72hrs incubation revealed that no detectable infectious particles had been generated by the fibroblasts as no production of the viral protein hexon could be detected in the cells (Fig 5H). These data therefore indicated that encoding complex therapeutic genes does not alter the selectivity or the activity of the enadenotucirev virus. Furthermore, this activity and tumour selectivity can be conferred to transgene expression.

It was also important when considering the platform as a tool to generate therapeutic products that virus manufacturing yields would not be affected by the introduction of multiple exogenous genes. Comparison of enadenotucirev and NG-135 virus particle yields was therefore carried out in HEK293 manufacturing cell line cultures both at small scale in culture shake flasks and at larger scale in a 5L bioreactor. Assessment of virus particle yields by HPLC analysis during small scale culture revealed no significant difference between enadenotucirev and NG-135 (Fig 5I) and yields post purification from the bioreactor were equivalent to GMP manufactured batches carried out previously for enadenotucirev. When the NG-135 virus was amplified in the bioreactor a high yield of antibody was also produced in the culture supernatant which was purified away from the virus material during the downstream manufacturing process and used for analysis of the antibody product by Biacore. Comparison of the virus-produced antibody to the clinically approved anti-VEGF antibody (bevacizumab) revealed equivalent functional properties in terms of VEGF binding with KD values of 2.49e-10 M and 1.73e-10M respectively (Fig 5J).

### Systemic delivery and local antibody expression in *in vivo* models

**Fig 6:**
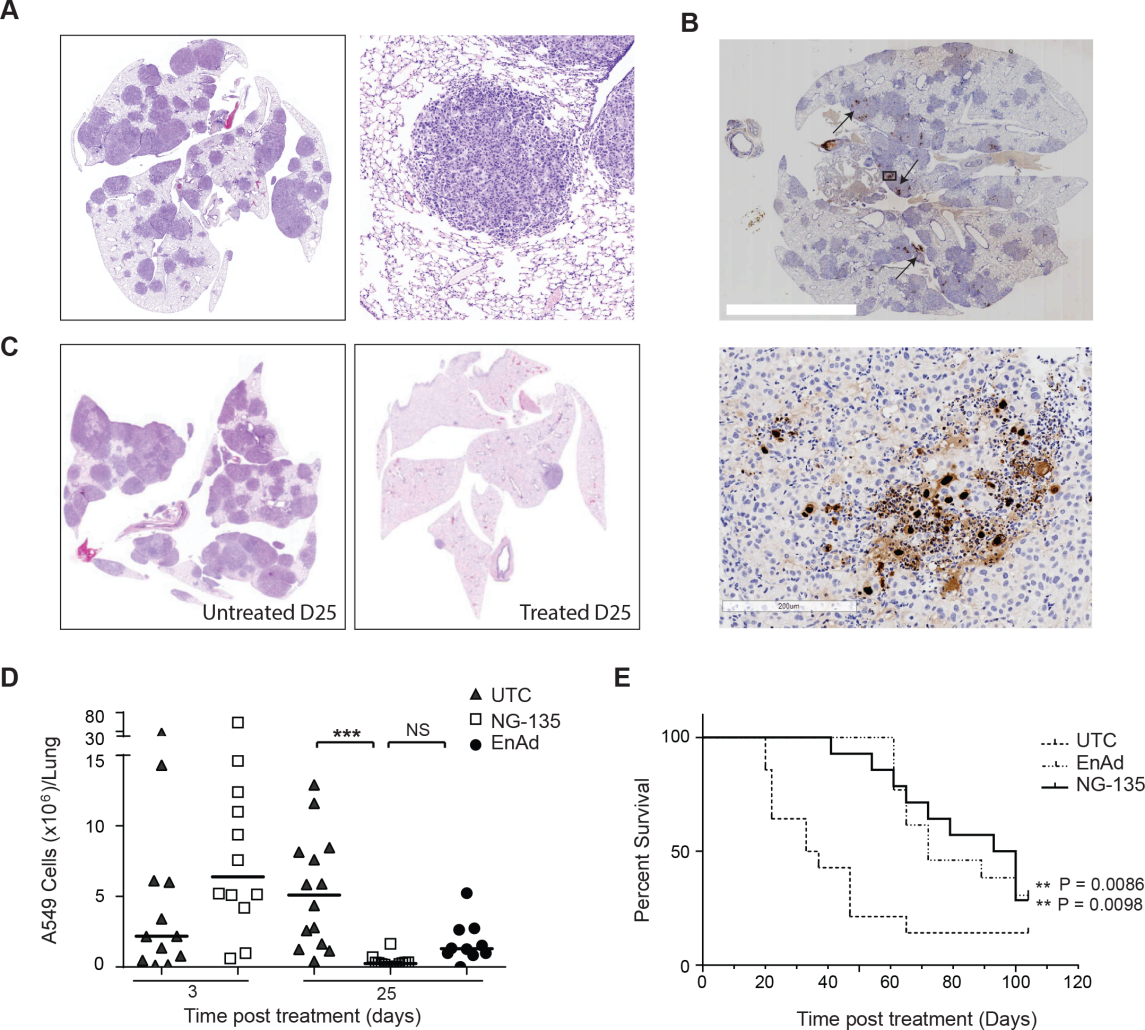
Antibody expression and virus activity in *in vivo* models following IV delivery. (A) Representative immunohistochemical staining of mouse lungs showing disseminated lung tumour burden 8 weeks post IV delivery of human A549 lung carcinoma cells (left panel) and a zoomed image of a tumour nodule (right panel). (B) Representative IHC images of virus late gene, hexon, detection in the lungs of a mouse bearing orthotopic xenograft A549 tumour nodules, at day 25 post IV treatment with 5x10^9^ virus particles. The left panel shows a section through the lungs, arrows indicate representative regions of hexon protein staining. The right panel shows a zoomed image of hexon protein stain within a tumour nodule. (C) Representative IHC images of tumour nodules in the lungs of mice 25 days post treatment with enadenotucirev virus particles (right panel) or mice that had not been treated by virus (left panel) (D) Quantification of total human A549 cells per lung by human cell line specific RTqPCR at days 3, 11, 18 or 25 post-IV treatment with NG-135 or enadenotucirev virus particles. Each data point represents the cell burden in a mouse lung (N>6 mice/group, ***P<0.005 one way ANOVA). (E) Kaplan-Meier plot showing percent survival for mice bearing A549 orthotopic xenograft tumours treated IV with 5x10^9^ NG-135 or enadenotucirev virus particles, or left untreated at day 0 (n = 10–12 mice per treatment group, ** P<0.005 Log rank test).

Having demonstrated *in vitro* that expression of functional antibodies was compatible with enadenotucirev activity, we set out to clearly understand the potential of enadenotucirev as a platform for local drug production following systemic delivery. We therefore set up a xenograft orthotopic lung cancer model using the human A549 lung carcinoma cell line. This model developed disseminated tumour nodules throughout the lungs (Fig 6A) and could therefore be used to investigate IV delivery of enadenotucirev or NG-135 to multiple tumour sites, as well as the oncolytic activity and therapeutic efficacy of the treatment. Following a single IV dose of virus (5x10^9^ vp), virus activity in the lungs was assessed by immuo-histochemical (IHC) detection of the viral capsid protein, hexon or detection of tumour nodules by H&E staining (Fig 6B and 6C). Viral activity could be detected at day 6 and day 25 post treatment as plaques of viral protein expression observable in multiple disseminated tumour nodules (Fig 6B) and viral efficacy by a reduction in the number of detectable tumour nodules remaining in the lungs at Day 25 (Fig 6C). To provide quantifiable assessment of these observations, tumour burden was measured by analysing the number of A549 cells in the lungs using a human cell line specific qPCR. Using this method, tumour burden was analysed over time (Day 3, 8, 11 and 25) post-treatment with NG-135 and revealed a steady reduction in tumour burden at each time point post treatment (S4 Fig). Comparison of tumour burden in NG-135 and enadenotucirev treated mice to untreated mice at Day 25 post-treatment showed both viruses significantly reduced tumour burden, with a >90% drop in total cell burden per lung in all NG-135 treated mice (Fig 6D). Although there was a trend at this time point for NG-135 treated mice to show a greater decrease in tumour burden than enadenotucirev treated animals, this difference was not statistically significant. The similarity of enadenotucirev and NG-135 activity in this model was not surprising as the anti-VEGF antibody encoded in the NG-135 virus is specific to human VEGF and would be unlikely to mediate direct anti-vascular effects in mouse models. However, these data do show that virus activity following IV delivery is not hindered by the inclusion of transgene. This data was confirmed by assessing enadenotucirev or NG-135 efficacy in this model by monitoring survival in a separate study. Mice were treated with a single, clinically relevant, IV dose of either enadenotucirev or NG-135 or left untreated once significant tumour burden had established (8 weeks post-A549 cell injection). Supportive of the tumour burden measurements assessed by qPCR both virus treated groups showed significantly improved survival compared to untreated controls and NG-135 virus efficacy was not impacted when compared to enadenotucirev (Fig 6E).

## Discussion

Oncolytic viruses with therapeutic proteins encoded in their genomes have the potential to overcome some of the challenges associated with treating patients with combinations of immunotherapeutic agents. Enadenotucirev has a number of unique features that indicated it could act as an effective platform for the delivery of therapeutic transgenes to tumours. These include its packaging capacity for exogenous genes resulting from innate genome deletions, its selective oncolytic activity in tumour cells and its ability to be delivered to tumours systemically. Here we have described the development of a molecular cloning system for directly inserting transgene cassettes encoding therapeutic proteins into the genome of enadenotucirev. This efficient method of cloning enabled the construction of a library of reporter viruses which was used to optimise the platform such that enadenotucirev’s viral characteristics are maintained despite functional transgene expression. Importantly, these studies indicated that this novel cloning platform can readily be used to generate panels of candidate viruses for a given therapeutic target as an early pre-clinical optimisation step. This cloning approach would therefore permit broad application of the enadenotucirev platform as a drug delivery system.

The majority of oncolytic viral platforms developed have used exogenous promoters to mediate transgene expression, for example T-Vec has GM-CSF encoded in its genome under the control of a CMV promoter [40]. The use of exogenous promoters either limits delivery to intra-tumoural dosing, to prevent off-target expression of the transgenes, or requires that the transgenes encoded have minimal off-target toxicities. This is because any cell that takes up a virus particle bearing a transgene under exogenous promoter control maybe transfected and thereby enabled to produce the transgene even in the absence of viral activity or infection.

Here we used the reporter virus library to show that for enadenotucirev-based viruses the endogenous major late promoter was able to mediate levels of transgene expression at equivalent or even higher levels to those using exogenous promoters such as CMV. These data therefore demonstrated that transgene expression could be effectively linked to the activity and selectivity of the enadenotucirev virus particle, avoiding the use of exogenous promoters. Significantly, these data translated to the functional expression of larger more complex proteins and the expression of multiple genes, such as the production of functional therapeutic IgG antibodies. Regardless of the complexity of the transgenes being expressed under the major late promoter enadenotucirev’s selective oncolytic activity in tumour cells, yields from manufacture and efficacy in tumours were all maintained. *In vivo*, transgene-encoding viruses could also be delivered to tumours by intravenous dosing and demonstrated functional activity without mediating off-target transfection and transgene expression in healthy body cells exposed to circulating viral particles (e.g liver cells).

Collectively these data demonstrated that enadenotucirev can be used as a versatile platform for encoding a wide range of different biopharmaceutical transgenes, which can be systemically delivered to mediate selective local production of therapeutic agents within the tumour microenvironment. This platform therefore has the potential to provide a single agent solution to some of the toxicity and cost challenges associated with treating cancer patients with systemically delivered immunotherapies. Importantly, this could be applicable to both single agents with serious adverse event profiles that have limited their ability to be dosed efficaciously or as a method to overcome the cumulative toxicities associated with systemic delivery of two or more immunotherapies.

## Materials and methods

### Cell lines and reagents

A549 (ATCC–CCL-185), HCT-116 (ATCC–CCL-247), SKOV (ATCC–HTB-77), HT-29 (Ark Therapeutics), DLD-1 (ATCC-CCL-221), AD-293 (Agilent Technologies), MRC-5 (ATCC–CCL-171), and Wi38 (ATCC–CCL-75) cell lines were maintained in DMEM high glucose with glutamine (Lonza) supplemented with 2mM L-glutamine (Gibco), 1mM Sodium pyruvate (Gibco) and 1mM non-essential amino acids (Gibco). For routine cell culture media was supplemented with 10% FBS (Gibco) and for assays with 2% FBS and 1mM Pen/Strep (Gibco).

### Vector generation

The plasmid pColoAd2.4 was obtained by homologous recombination between a shuttle vector, pColoAd2.4 Shuttle, and the enadenotucirev genome. The detailed construction of the pColoAd2.4 plasmid was as follows: A ~12kb shuttle plasmid, pColoAd Shuttle, was constructed in order that unique restriction sites could be introduced in the late gene, L5, region of the enadenotucirev genome. The 5’ (nt 1–4632) and 3’ (nt 27837–32326) ends of enadenotucirev were amplified from the enadenotucirev genome by PCR using the primer 5’–TTGGCGGCGCGCCTATCTATATAATATACC-3’ and primers 5’-AATGCAAATCTGTGAGGGG-3’ or 5’–CTTAGTGGTGTTGTGGTATTGG-3’ respectively. The 5’ arm PCR product contained an introduced 5’ AscI site and 3’ PspOMI site that corresponds to the PspOMI site at nt 4626 in the enadenotucirev genome. The 3’ arm PCR product contained a 5’ PspOMI site that corresponds to the PspOMI site at nt 27837 in the enadenotucirev genome and a introduced 3’ AscI site. The PCR products were restriction digested with AscI/PspOMI and ligated in a one-step three-way ligation into an AscI linearised plasmid that contained a p15A origin of replication and a kanamycin resistance cassette. This generated the pColoAd1 Shuttle plasmid.

A DNA fragment corresponding to the region of the ColoAd1 genome that is flanked by PspOMI and AclI restriction sites and contains the late gene, L5, (nt 27837–30060) was synthesised with an added region of 19bp 5’- GCGATCGCTACCCTGCAGG-3’ inserted at position corresponding to enadenotucirev nt 29355 (Mwg-Eurofins). This additional region included restriction sites for two enzymes that are not present in the enadenotucirev genome, AsiSI and SbfI. The synthesised DNA fragment was restriction digested with the enzymes PspOMI and AclI and cloned into the corresponding region in the PspOMI/AclI digested pColoAd1 shuttle plasmid to create the plasmid, pColoAd2.4 shuttle. To obtain the pColoAd2.4 plasmid by homologous recombination the pColoAd2.4 shuttle plasmid was linearised by restriction digest with the enzyme PspOMI and treated with alkaline phosphatase to remove 5’ phosphates. The linearised plasmid and the enadenotucirev genome were co-transfected into BJ5183 cells by electroporation according to the manufacturer’s protocol and the generation of the pColoAd2.4 plasmid by homologous recombination was determined by restriction digest.

The plasmid pColoAd2.6 was synthesized directly by Gibson Assembly using nucleotide sequence information (SGI DNA, La Jolla, CA).

Correct construction of all plasmids was confirmed by DNA sequencing (MWG-Eurofins, Germany).

### Transgene cloning

Transgene cassettes encoding eGFP, luciferase, or anti-VEGF antibody were generated synthetically by MWG-Eurofins with flanking 5’ AsiSI and 3’ SbfI restriction sites. The plasmids pColoAd2.4 or pColoAd2.6 were linearised by restriction digest with AsiSI and SbfI for 2hrs, 37°C, and then gel purified for subcloning using the QIAEX II DNA purification kit (Qiagen, UK) according to the manufacturer’s protocol. 120ng of AsiSI/SbfI digested transgene cassette was ligated into 60ng of linearised pColoAd2.4 or pColoAd2.6 using 2μL T4 DNA ligase for 16 hrs at 16°C. One third volume of the ligation reaction was transformed into competent *E*.*coli* (XL Blue, Agilent Technologies, Santa Clara, CA) according to the manufacturer’s protocol. Colonies were selected on kanamycin LB agar, amplified overnight and the DNA purified by miniprep (Qiagen, UK). Plasmids containing inserted transgenes were screened by restriction digest and confirmed by sequencing (MWG-Eurofins, Germany).

### Virus production, purification and titration

7μg of pColoAd2.4 or pColoAd2.6 plasmids containing transgene cassettes encoding eGFP, Luciferase or anti-VEGF antibodies were digested with AscI to excise the linear viral genomes. Genomes were purified for transfection by phenol:chloroform extraction and overnight ethanol precipitation. The day before transfection, 4x10^6^ HEK293 cells were seeded in to T-25 culture flasks. Transfection was carried out using ~5μg of DNA and 15μL of GeneJuice transfection reagent (Millipore, UK) in 1mL of OptiMEM. Culture media was added to the cells 2hrs post transfection and the cells were then observed every 24hrs until significant CPE occurred. The virus particles were harvested from the cells by freeze-thaw and the virus was amplified on HEK293 before purification by double caesium chloride banding [41]. For viral amplification in HEK-293 suspension cells, the cells were thawed and expanded in shake flasks. For small scale yield tests, 125mL shake flasks were seeded at a density of 1x10^6^ cells/mL and infected with an MOI of 50 ppc for 40, 46, 49 or 64 hrs before virus particles were harvested by freeze-thaw. For bioreactor amplification, HEK293 cells were expanded to a 3 L working volume in a 5 L stirred-tank bioreactor with parameters of 37°C, pH 7.4, dissolved oxygen of 50, airflow rate of 100mL/min and agitation at 100 rpm. The culture was infected with NG-135 at an MOI of 50ppc for 48 hrs before purification using a process previously established for GMP manufacture of enadenotucirev. The anti-VEGF antibody product produced was also purified from the harvested material using tangential flow filtration followed by Protein A chromatography (ÄKTA Protein Puriifcation systems, GE Healthcare).

Virus titre was quantified using a ratio of optical density at 260 (DNA) /280 (Protein) and High Performance Liquid Chromatography (HPLC). For optical density quantification the viral particle concentration was calculated using an absorbance of 1.00 AU (1 cm pathlength) at 260 nm corresponding to 1.1x10^12^ viral particles/mL. For HPLC quantification a Resource Q (anion exchange) column was used and virus elution detected at 260nm. The concentration of virus was determined by integrating the 260 nm signal of the virus elution peak and calculating the concentration from an enadenotucirev standard curve.

### Virus activity assays

Unless otherwise stated tumour cell lines were seeded in 12 well plates at a density of 3.5x10^5^ cells/well for A549, 1x10^6^ cells/well for HT-29, 1x10^6^ cells/well for HCT-116, 3.5x10^5^ cells/well for DLD, 2x10^5^ cells/well for SKOV and incubated overnight before infection with virus particles in assay media supplemented with 2% FBS.

#### Quantification of viral genomes or A549 cells by quantitative PCR (qPCR)

Total DNA was extracted from cell lysates or culture supernatants using the GenElute mammalian genomic DNA extraction kit (Sigma Aldrich, US) and following the ‘cell lysates’ manufacturer’s protocols. Total DNA was extracted from tumour and tissue homogenates using the DNeasy Blood and Tissue kit (Qiagen, UK) according to the manufacturer’s protocol. For assessing viral genomes, a six point, tenfold standard curve was generated using enadenotucirev virus particles spanning 2.5x10^3^vp to 2.5x10^8^vp spiked into the relevant assay matrix. The primers and probe were as follows; forward primer: 5’–ATCCATGTCTAGACTTCGACCCAG– 3’, reverse primer: 5’—TGCTGGGTGATAACTATGGGGT– 3’ Probe: 5’—FAM-ATCTGTGGAGTTCATCGCCTCTCTTACG-TAMRA– 3’. For analysing A549 cells human specific sequences targeting the human prostaglandin E receptor 2 (PTGER2) gene region were used. This primer probes set has been previously characterised in Alcoser et al [42] and allow quantification of human tissue in murine xenograft models. DNA samples were analysed on the StepOne Plus Real time PCR machine (Thermo Fisher Scientific) in a 10μL reaction volume containing 2μL of DNA, 8μL of Taqman Fast advance reaction mix, 80nM of each primer and 20nM of probe. The initial denaturation step at 95°C for 20 sec was followed by 40 cycles of 95°C for 1 second and 60°C for 20 seconds.

#### Cell Viability assay to assess virus potency

HT-29 cells were seeded at 2.5x10^4^ cells/well in 96 well plates and incubated for 4–6 hrs. Cells were infected in triplicate with one of 9 dilutions of virus in a range of 0.39ppc to 100ppc. After 72 hrs Cell Titer 96 Aqueous One Stock Solution MTS Reagent (Promega) was added to the cells and incubated for 30 mins. Absorbance readings at 490nm were aquired using a Synergy HT Plate Reader (BioTek).

#### Quantification of GFP expression in tumour cells

Cell lysate samples were harvested 24, 48, 72 and 96 hours post infection. Samples were added to black 96 well plates neat or 1:2 diluted in lysis buffer and fluorescence measured using the Synergy HT Plate Reader (Biotek).

#### Quantification of luciferase expression in tumour cells

Luciferase activity was determined in virus infected cells using the using the BrightGlo Luciferase assay system (Promega) according to the manufacturer’s instructions. Samples or recombinant luciferase controls and standards were analysed using a Synergy HT Plate Reader (Biotek).

#### Quantification of functional antibody expression by ELISA

Media was removed from infected or control cells, clarified by centrifugation and diluted 1 in 2 in 3% BSA/PBS for storage at -20°C. For quantification of human IgG1, 96 well plates (NUNC) were coated overnight, 4°C, with mouse monoclonal anti-human IgG1 Fc antibody (ab1927, Abcam) at a 1:1000 dilution in carbonate/bicarbonate buffer. Plates were blocked in 3% BSA/PBS, washed and samples and standards added to the plate for 1 hr and RT. Secondary detection was carried out using a HRP conjugated goat anti-human IgG Fab (ab87422, Abcam) incubated for 1 hr, RT. For quantification of anti-VEGF antibody expression, 96 well plates were coated overnight, 4°C, with recombinant human VEGF-165 (0.5 μg/mL, R and D Systems, 293-VE-050). Plates were blocked in 3% BSA/PBS and samples and standards added to the plate for 1hr, RT. Secondary detection was carried out using HRP conjugated goat polyclonal anti-Human IgG-Fc HRP (Abcam, ab97225) diluted to 1:100000. For both ELISAs washes were performed between each step with 0.05% PBS Tween-20 and Ultra TMB-ELISA substrate (Sigma) was incubated with the plates for 30 mins then 1M HCL added before plates were read.

#### Assessment of virus activity in fibroblast cell lines

HT-29 (1.5x10^6^ cells/well), MRC-5 or Wi38 cells (3.75x10^5^ cells/well) were seeded in 12 well plates for 4–6 hrs before infection with 100ppc. After incubation with virus for 4 hrs, the media was replaced in the wells and plates incubated for 1, 12, 24, 48 or 72 hrs. At each time point, cells and clarified cell media were harvested for qPCR and ELISA (see above methods). Infectious virus particle content of the cell media was assessed by TCID50 assay. For this HT-29 cells were seeded at 3x10^4^ cells/well in 96 well plates and incubated for 24 hrs. Cells were infected in quadruplet with supernatant diluted over a six point five-fold serial dilution from neat. After 70 hrs cells were fixed using 1:1 Methanol:Acetone at RT for 10 mins, air dried and then washed with PBS. Virus was detected using 100μL rabbit anti-Adenovirus antibody (B025/AD51, Abcam) added for 60 mins at RT. Plates were washed twice with PBS and secondary detection was carried out using 100μL 1:800 HRP conjugated Rabbit polyclonal anti-mouse IgG H+ L (Abcam). Plates were washed twice with PBS and virus visualised using 60μL 1:50 DAB (DAB substrate kit, Abcam). Plates were washed and wells containing stained cells counted. The effective titre was calculated using the Spearman-Karber formula: Virus Titre (TCID50/ml) = 10^[a + 1.5 + xa/8 + xb/8 + xc/8]^. Where: a = dilution factor of last column in which 8 wells are positive. xa = number of positive wells in column a + 1. xb = number of positive wells in column a + 2. xc = number of positive wells in column a + 3.

#### Biacore analysis

VEGF165 (ligand) was directly immobilized onto the C1 sensor chip with a defined contact time and flow rate using amine coupling. A concentration series of bevacizumab (analyte) was passed over the surface of immobilized VEGF165 to determine the kinetics of binding. Reference standard and samples were injected and regeneration cycles were set following a defined protocol in Biacore T200 control software. The kinetic analysis was performed with Biacore T200 Evaluation Software.

### *In vivo* assessment of virus activities

All animal experiments were performed in accordance the Animals (Scientific Procedures) Act 1986 and all work was approved by the Oxford animal care and ethical review board (ACER). Where anaesthesia was carried out isoflurane was used and euthanasia was carried out by cervical dislocation with confirmation by exsanguination. All mice were obtained from Charles River and were acclimatised for 1 week before studies were commenced.

For sub-cutaneous tumours 2x10^6^ A549 tumour cells were injected sub-cutaneously into the flank of SCID mice in 50μL PBS. Once tumour volume reached ~100mm^3^, mice were dosed either IV (100μL) or IT (10μL) with 5x10^9^ vp in PBS. Tumour volume was measured 3–4 times per week until either a pre-defined study endpoint or a humane endpoint was met. Survival readouts utilised tumour volume as a humane endpoint, where tumour volume was restricted to 1200mm^3^.

For the A549 orthotopic lung model, 6x10^5^ A549 tumour cells were implanted intravenously into SCID mice on two consecutive days in 200μL PBS. Mice were randomised between treatment groups and then dosed IV with 100μL PBS containing 5x10^9^ vp 6–7 weeks after implantation. Mouse weight was measured 4 times per week until either a pre-defined study endpoint or a humane endpoint of 10% weight loss relative to peak weight was reached. Weight loss was used in survival studies as the humane endpoint.

For imaging luciferase activity, mice were anaesthetised with isoflurane before being injected IP with 200μL luciferin potassium salt (GoldBio) at 15.8 mg/mL. Mice were imaged 6 mins post injection using an IVIS 100 camera (PerkinElmer). Regions of interest of a fixed size were used across each study to determine relative light units (photos/second/cm^2^) in the tumour and liver.

#### Tumour harvest and homogenisation

Mice were sacrificed and tissues or tumours were resected, snap frozen in liquid nitrogen and stored at -80°C. Samples were homogenised in reporter lysis buffer (Promega) at 250mg/mL using a FastPrep-24 benchtop homogeniser (MP Biomedicals). Tissue or tumour lysates were used for quantification of virus genomes by extracting total DNA and carrying out qPCR analysis (as described above), for assessment of transgene expression by ELISA or for assessment of virus particle activity by re-infection assay (described below). Where tissues were to be analysed by ELISA, sample collection and homogenisation was as described above with the exception that the reporter lysis buffer was supplemented with 1 in 200 protease inhibitor cocktail III (Calbiochem). 100μL of each tissue homogenate was diluted 1 in 2 with reporter lysis buffer containing 2% Triton X (Sigma) before sonication in a water bath for 3 minutes. 0.5μL per sample of benzonase was added to each sample which were then incubated at room temperature for 20 mins before being diluted 1 in 2 in assay buffer and carrying out the anti-human IgG1 ELISA.

#### IHC for virus hexon (and H&E)

Subcutaneous tumours were resected and immediately submerged in 5mL formalin solution (Sigma). For mice implanted intravenously, lungs were perfused with formalin solution before tying off the trachea with suture thread and submerging in 5ml formalin solution. All tissues were left at ambient temperature in the dark for 24 hours before being washed with 5mL of 70% EtOH and submerged in 5mL of fresh EtOH. Tissues were embedded in paraffin and cut into 4μm sections before staining with either hematoxylin and eosin or an anti-hexon monoclonal antibody (AbCam clone 1E11).

### Statistical analysis

Column statistics were performed with GraphPad software (Prism Version 6). Mean values and SD are shown unless indicated otherwise. Two sample groups were analysed by *t* test. Groups larger than 2 were analysed by 1-way ANOVA. For parametric data, a Bonferri posttest was applied and for nonparametric data a Kruskal-Wallis test was carried out followed by Dunn multiple comparison posttest. Survival distributions were compared using a log-rank test.

## Supporting information

S1 Fig Kinetics of genome replication and GFP expression in NG-106 or NG-108 infected cells. (A) The total genomes generated per HT-29 colon carcinoma cell infected for 24–96 hrs with 1 particle per cell (ppc) NG-106 or NG-108 virus particles. Graph shows mean ± SD (n = 3 independent experiments). (B) Quantification of GFP fluorescence in HT-29 cells infected for 24-96hrs with 1ppc of NG-106 or NG-108 virus particles. Graph shows the fold change in fluorescence at each time point relative to uninfected control cells plotted as mean ± SD (n = 3 independent experiments).
(TIF)

S2 Fig Virus-mediated luciferase transgene expression in A549 xenograft tumours. Bioluminescence heat map images of mice imaged day 2 –day 49 post-treatment with NG-285 delivered by IT (left panel) or IV (right panel) administration. At day 49 post-treatment one of the mice treated by IT delivery had reached its survival endpoint (tumour volume >1200mm^3^) and so was not able to be imaged.
(TIF)

S3 Fig Schematic of the transgene cassette in the NG-135 virus. (TIF)

S4 Fig Reduction in A549 orthotopic tumour burden following IV treatment with NG-135 virus. Quantification of total human A549 cells per lung by human cell line specific RTqPCR at days 3, 11, 18 or 25 post-IV treatment with NG-135 virus particles. Each data point represents the cell burden in a mouse lung (N>6 mice/group.
(TIF)
