# Supplementary figures and images for: Development of a versatile oncolytic virus platform for local intra-tumoural expression of therapeutic transgenes
Source: PLoS One. 2017 May 18;12(5):e0177810. doi: 10.1371/journal.pone.0177810 (PMC5436815; doi:10.1371/journal.pone.0177810)

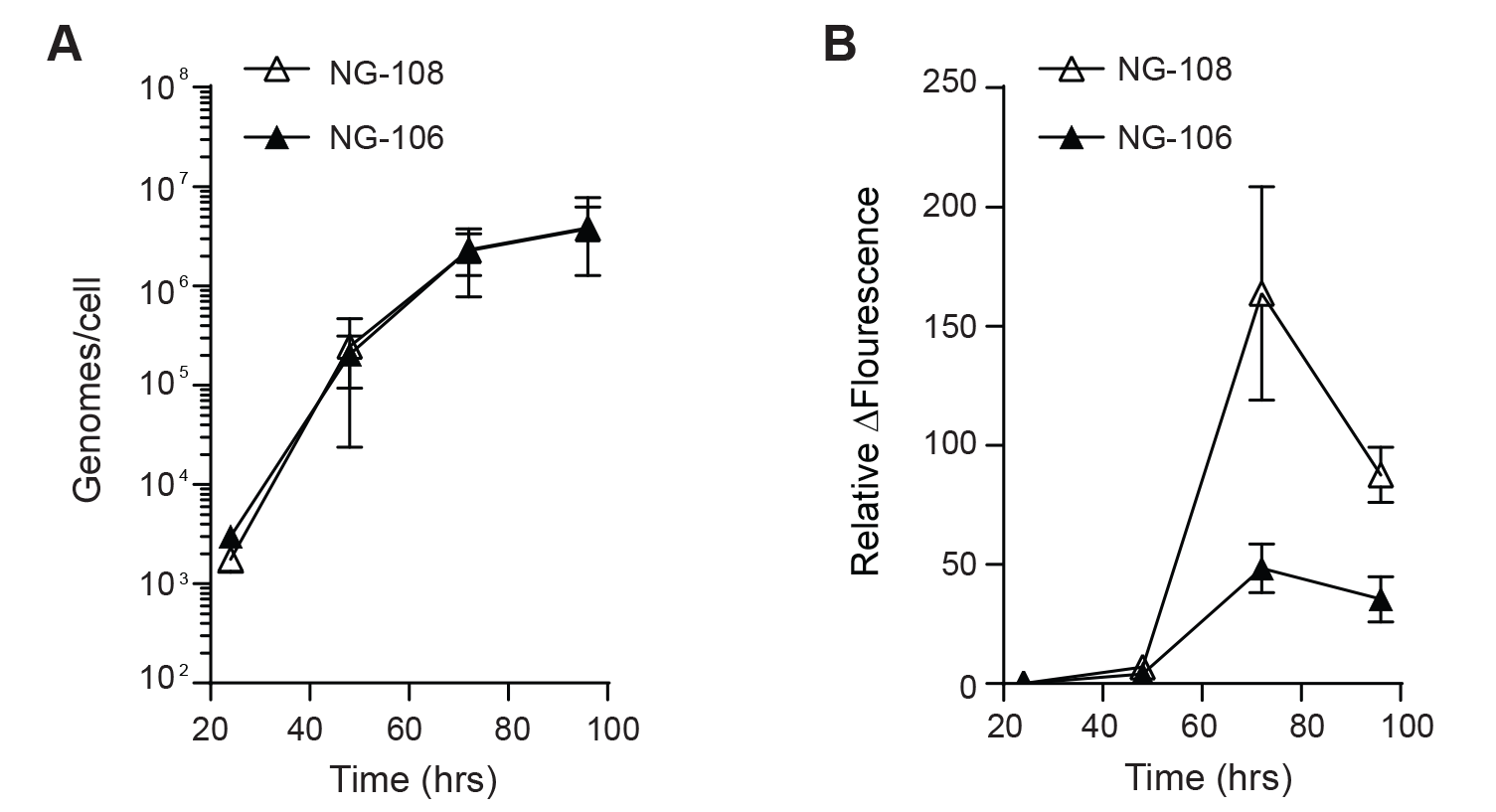

Supplement: S1 Fig — (A) The total genomes generated per HT-29 colon carcinoma cell infected for 24–96 hrs with 1 particle per cell (ppc) NG-106 or NG-108 virus particles. Graph shows mean ± SD (n = 3 independent experiments). (B) Quantification of GFP fluorescence in HT-29 cells infected for 24-96hrs with 1ppc of NG-106 or NG-108 virus particles. Graph shows the fold change in fluorescence at each time point relative to uninfected control cells plotted as mean ± SD (n = 3 independent experiments). (TIF) [file pone.0177810.s001.tif]

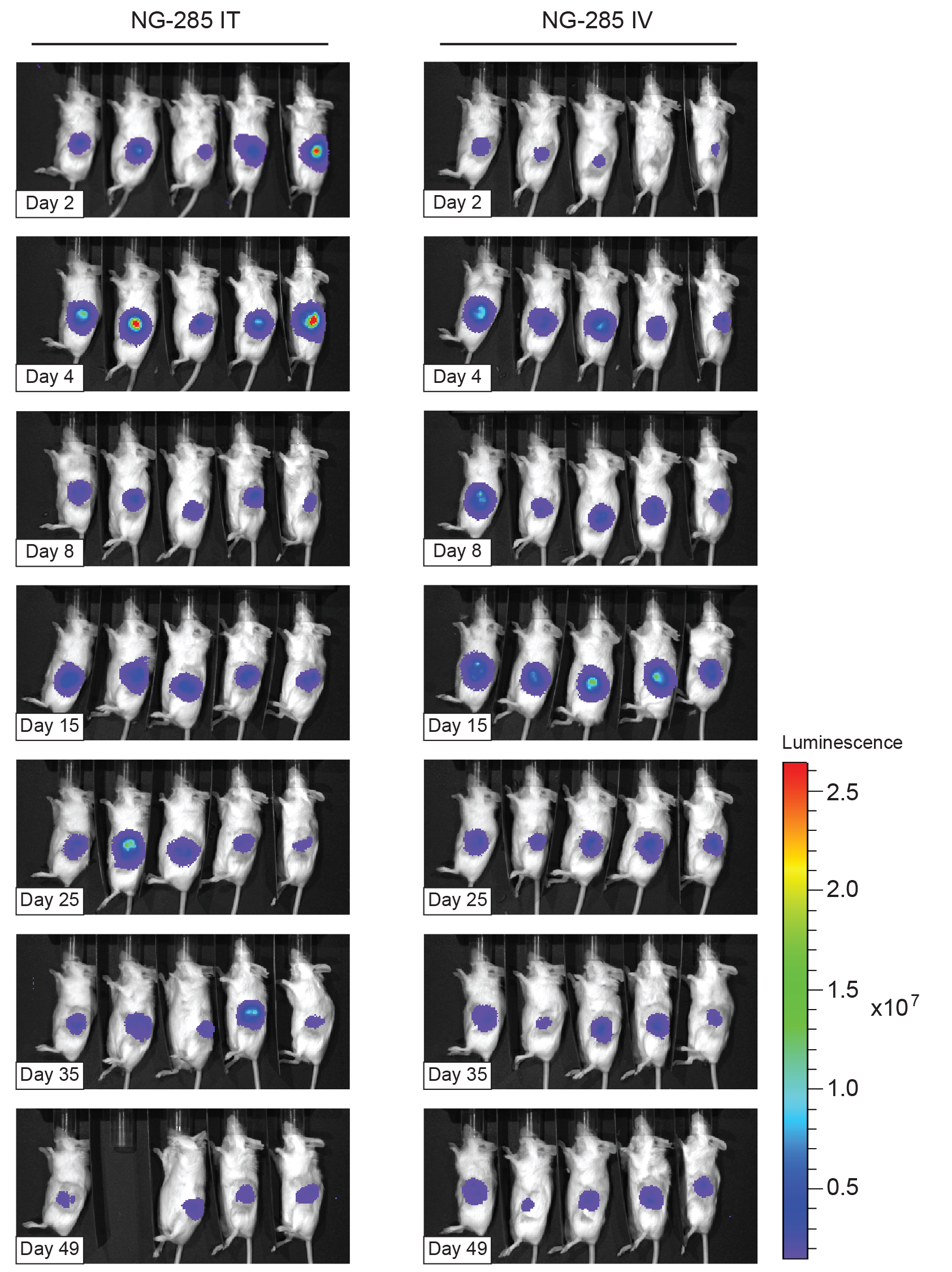

Supplement: S2 Fig — Bioluminescence heat map images of mice imaged day 2 –day 49 post-treatment with NG-285 delivered by IT (left panel) or IV (right panel) administration. At day 49 post-treatment one of the mice treated by IT delivery had reached its survival endpoint (tumour volume >1200mm3) and so was not able to be imaged. (TIF) [file pone.0177810.s002.tif]

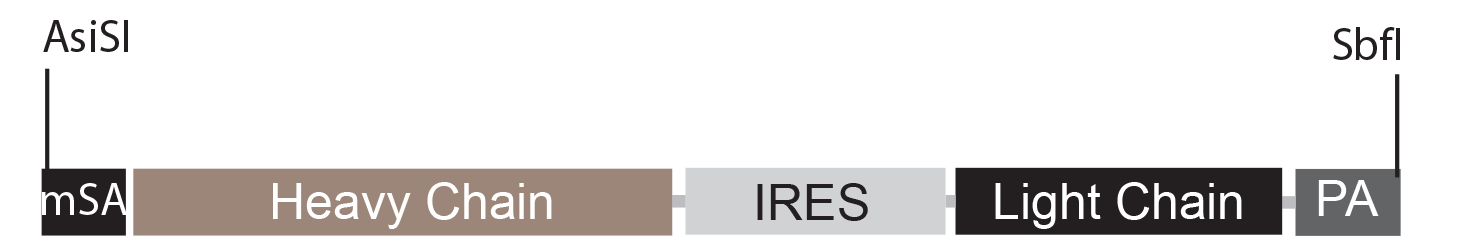

Supplement: S3 Fig — (TIF) [file pone.0177810.s003.tif]

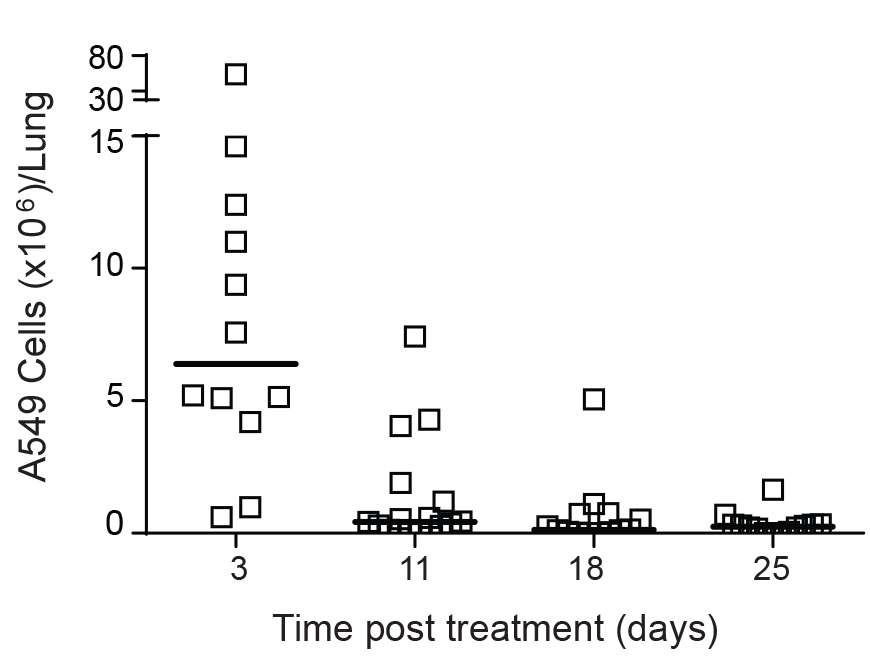

Supplement: S4 Fig — Quantification of total human A549 cells per lung by human cell line specific RTqPCR at days 3, 11, 18 or 25 post-IV treatment with NG-135 virus particles. Each data point represents the cell burden in a mouse lung (N>6 mice/group. (TIF) [file pone.0177810.s004.tif]
